# Supplementary material for: Role of a Pdlim5:PalmD complex in directing dendrite morphology
Source: Front Cell Neurosci. 2024 Feb 13;18:1315941. doi: 10.3389/fncel.2024.1315941 (PMC10896979; doi:10.3389/fncel.2024.1315941)
Supplement: Supplementary file 1 [file Presentation_1.pdf]

SUPPLEMENTAL FIGURE TITLES & LEGENDS.

Supplemental Figure 1. Pdlim5 conservation and function.

A. The conservation of Pdlim5 among various vertebrates is depicted in the figure. Conserved sequences are represented in red, while identical sequences are shown in gray. Gaps in the alignment are denoted by red lines. The number of amino acids for each species is indicated in the top right corner, and the species names are listed on the left side. The multiple sequence alignment was conducted using Clustal Omega software.

B. Impact of Pdlim5 knockdown (siRNA992, siRNA993) on hippocampal neuron branching morphology. Exogenous expression of GFP (control), Pdlim5, and siRNA-mediated knockdown of Pdlim5. Following siRNA-mediated knockdown of Pdlim5 (siRNA992, siRNA993), we observed a significant reduction in dendritic complexity. In contrast, the exogenous expression of Pdlim5 resulted in an increase in branching morphology in comparison to negative control GFP. n=10 neurons each from three biological replicates.

C. Illustrative images demonstrating the knockdown (KD) of Pdlim5. The control GFP image (green, first image on the left) exhibits less branching compared to the expression of exogenous Pdlim5 (green, second image from the left), while loss of branching morphology is observed in the Pdlim5 knockdown siRNA images (green, third and fourth images from the left). Neuron health was assessed using the Map2 marker. n = 3 biological replicates. The scale bars in the images indicate 50 µm. See panel D for quantitation.

D. The Pdlim5 total cell fluorescence was quantified using ImageJ for negative control (GFP), positive control Pdlim5, and siRNA mediated knockdown of Pdlim5 (siRNA992, siRNA993). As expected in comparison to control, exogenous Pdlim5 significantly increases Pdlim5 total cell fluorescence while knockdown significantly decreases Pdlim5 total cell fluorescence. n≥10 neurons in each of three biological replicates. The quartiles represent data ranges and middle dark line represent median, and the statistical significance, determined using one-way ANOVA, is indicated as $P \leq 0.0001$ (****).

Supplemental Figure 2. Selected Pdlim5 associated candidates with known cytoskeletal functions.

Our yeast-2-hybrid (Y2H) screen suggested several Pdlim5-associated candidates involved in cytoskeletal functions. In total we resolved over 70 candidates with 11 being implicated in cytoskeletal contexts, including for example Beta-III Spectrin, Beta-IV Spectrin, and alpha-Actinin4. Our studies here focused upon PalmD for reasons outlined in the text.

Supplemental Figure 3. PalmD, a novel partner of Pdlim5, associates with Pdlim5's LIM domain.

A. The endogenous co-immunoprecipitation (co-IP) of Pdlim5 and PalmD was performed from rat primary cortical neurons at DIV9. Blots were used to demonstrate the pull-downs of Pdlim5 and PalmD. Separate blots for endogenous PalmD and Pdlim5 were undertaken given their similar molecular weights (~70kDa). Endogenous self-IP blots were also performed, confirming the effectiveness of the endogenous IP procedures. n≥3 biological replicates for each condition.

B. In exogenous contexts (HEK293 cells), we engineered Pdlim5 with a 6x-Myc-epitope tag (MT) at its Nterminus (~75kD), while PalmD was fused with GFP (~100kD). Together with the findings noted in Figures 2 and 3, and immediately above in Panel A, we collectively provide evidence of both endogenous and exogenous association between Pdlim5 and PalmD. n≥3 biological replicates in each condition.

C. PalmD co-immunoprecipitation with Pdlim5's isolated LIM domain. PalmD was co-expressed with each individually isolated domain of Pdlim5 (LIM or PDZ). The results revealed that PalmD associates specifically with the LIM domain of Pdlim5. Relative to negative controls, no association was observed with the other isolated domains, such as PDZ (or with the central DUF domain; not shown). n≥3 biological replicates in each condition. See also below Panel D.

D. In addition to using co-IP (see panel C above), we applied an orthogonal method we term GLS (see the text and Methods), to explore the association of different domains of Pdlim5 with PalmD. The assay revealed the colocalizations of PalmD with Pdlim5 at the Golgi only when the LIM domain of Pdlim5 was present. This finding again suggests that PalmD has an association with the LIM domain of Pdlim5. n≥3 biological replicates.

E. Calculation shows the Pearson's correlation coefficient for the co-distribution of Pdlim5 subdomains and PalmD in the presence GLS-tag upon Pdlim5 subdomains. The level of statistical significance is indicated as ****, which signifies a p-value of ≤ 0.0001 determined using one-way ANOVA. The violin plot quartiles represent data ranges, and the middle dark line represents the median.

Supplemental Figure 4. Function and functional dependency of Pdlim5: PalmD complex.
A. Imaris-generated 3D rendering image of rat primary hippocampal neurons shows the exogenous expression of negative control GFP, Pdlim5, or PalmD. Increased branching is observed in neurons expressing Pdlim5 or PalmD compared to the GFP control. Panel B provides quantitation of the average number of tips per cell. n≥3 biological replicates.

B. Imaris 9.9.0 and Image J software was utilized to quantify the average number of tips per cell. A box and violin plot illustrates significantly increased branching upon Pdlim5 or PalmD exogenous expression. Each dot represents a single neuron (n=10), and the levels of statistical significance, determined using one-way ANOVA, are indicated as P ≤ 0.0001 (****). n≥10 neurons.

C. A bar graph compares negative-control GFP, exogenous PalmD expression, and knockdown of PalmD (siRNA-714; siRNA-716) based on Imaris 9.9 tool analysis (see Methods). The analysis reveals that PalmD acts as a modulator for branching function. The loss of PalmD is associated with a decrease in branching morphology, while exogenous expression of PalmD enhances branching. Each dot represents a single neuron (n=10), and the levels of statistical significance, determined using one-way ANOVA, are indicated as P ≤ 0.0001 (****), n≥10 neurons each from three biological replicates.

D. Correlated total cell fluorescence was quantified using Image J software, indicating that knockdown of PalmD significantly reduces fluorescence intensity compared to the negative-control GFP or to positive-control PalmD-overexpressing neurons. $n \geq 10$ neurons in each of three biological replicates. Each dot represents a single neuron. The violin plot quartiles represent data ranges, the middle dark line represents the median, and the statistical significance, determined using one-way ANOVA, is indicated as $P \leq 0.0001$ (****).

E. Sholl analysis was performed to analyze dendrite morphology in neurons under different conditions: GFP control, exogenous PalmD expression, and exogenous PalmD expression in the presence of knockdown of Pdlim5 (siRNA-992). Neurons overexpressing PalmD exhibit a significant increase in dendritic complexity compared to GFP-expressing cells. However, when Pdlim5 siRNA-992 is introduced alongside PalmD exogenous expression, there is a significant loss of branching function. $n \geq 15$ neurons in each of three biological replicates. The significance was assessed using a two-way ANOVA with Bonferroni post-hoc analysis. The levels of significance varied from $P \leq 0.0001$ to $P \leq 0.05$, depending on the radial distance from the soma.,

F. Representative binary images (see Methods) indicate the interdependency of Pdlim5's and PalmD's branching functions. In the absence of Pdlim5, PalmD no longer enhances branching morphology. Analogously, in the absence of PalmD, Pdlim5 displays a loss of branching phenotype. n=3 biological replicates. Scalebars 20µm.
