# Supplementary figures and images for: Role of a Pdlim5:PalmD complex in directing dendrite morphology
Source: Front Cell Neurosci. 2024 Feb 13;18:1315941. doi: 10.3389/fncel.2024.1315941 (PMC10896979; doi:10.3389/fncel.2024.1315941)

Figure S1: Pdlim5 conservation and function

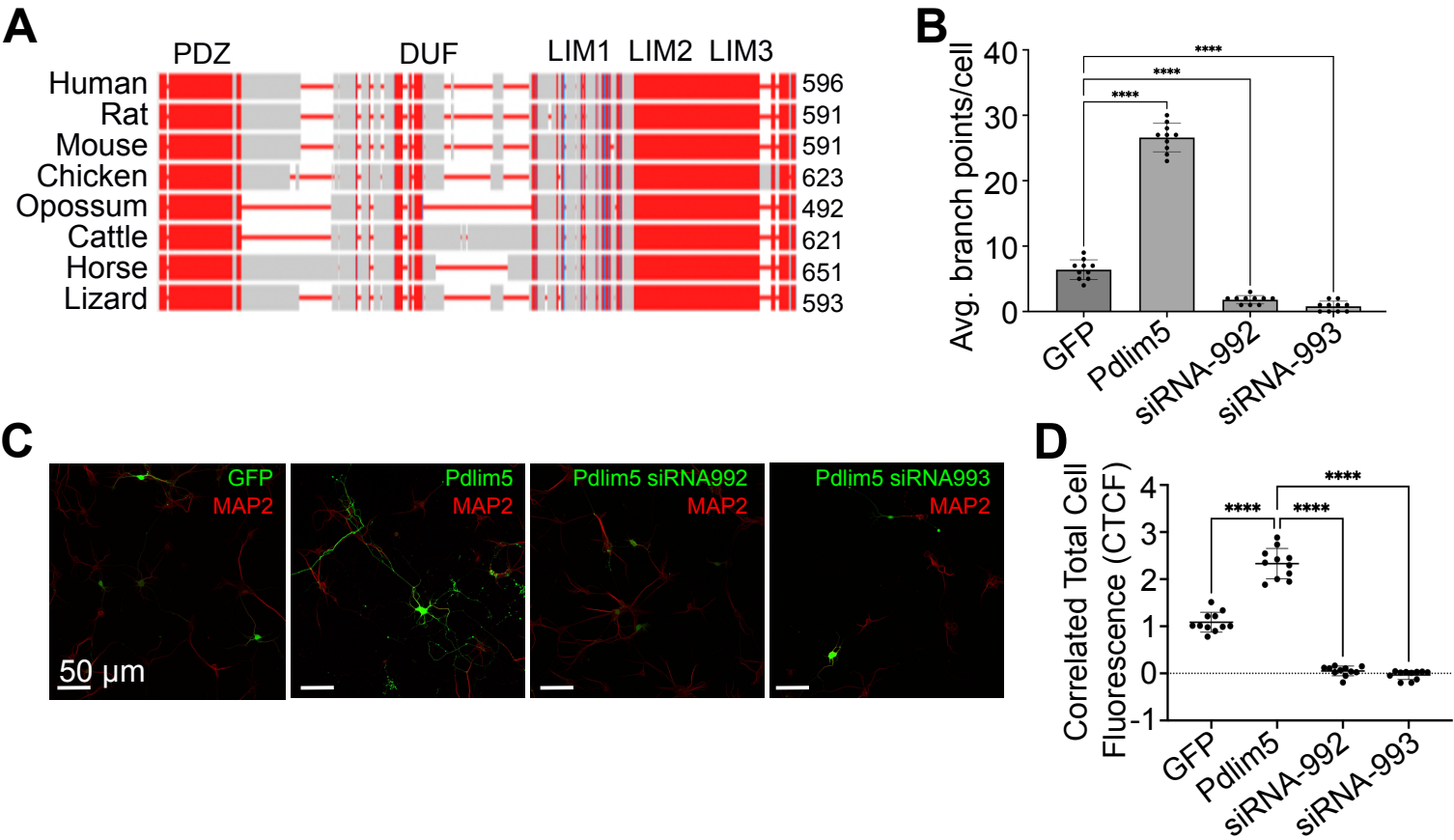

Supplement: Supplementary file 2 [file Image_1.pdf]

Figure S3: PalmD, a novel partner of Pdlim5, associates with Pdlim5's LIM domain

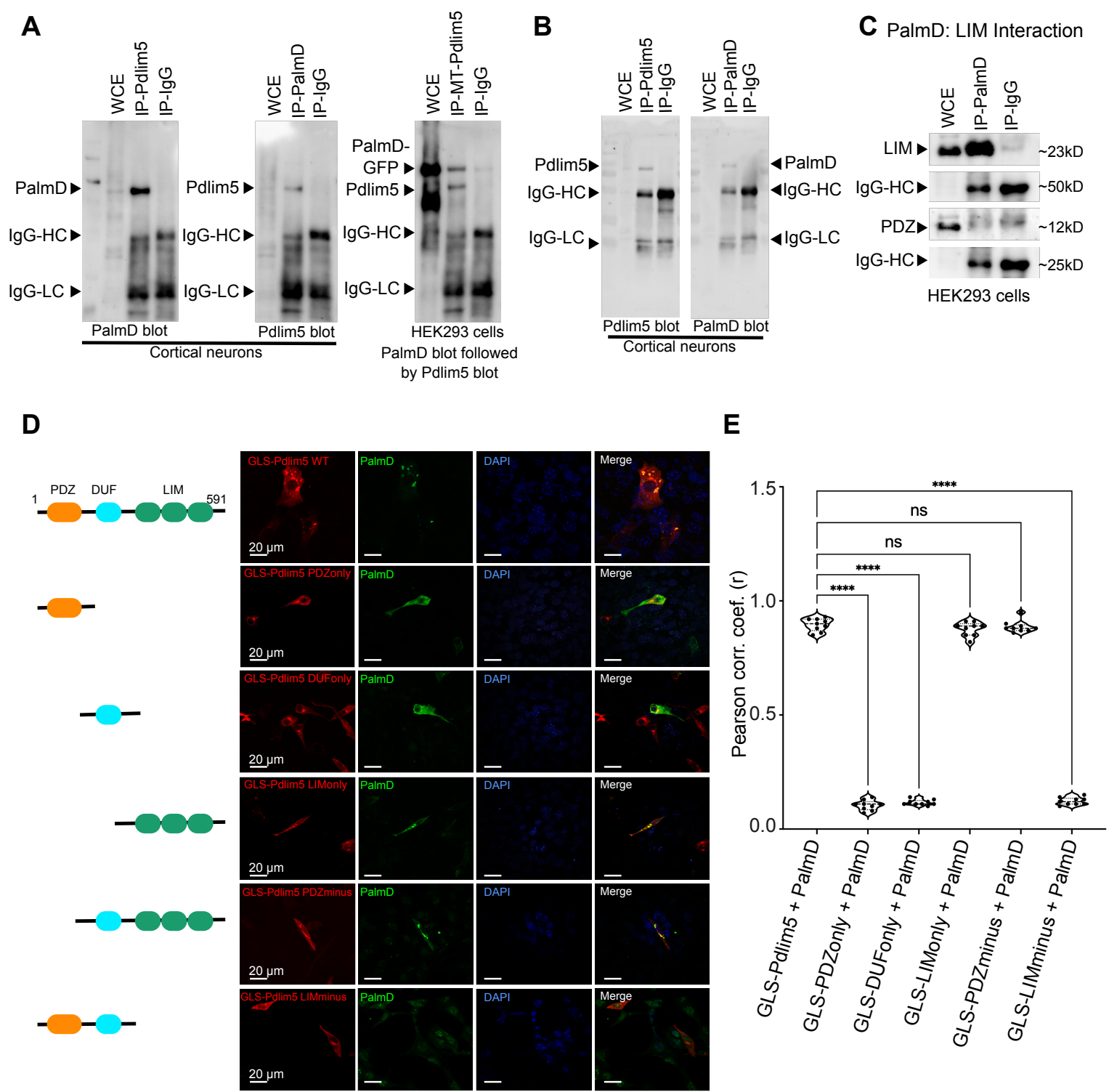

Supplement: Supplementary file 4 [file Image_3.pdf]

Figure S4: Function and functional dependency of Pdlim5:Palmd complex

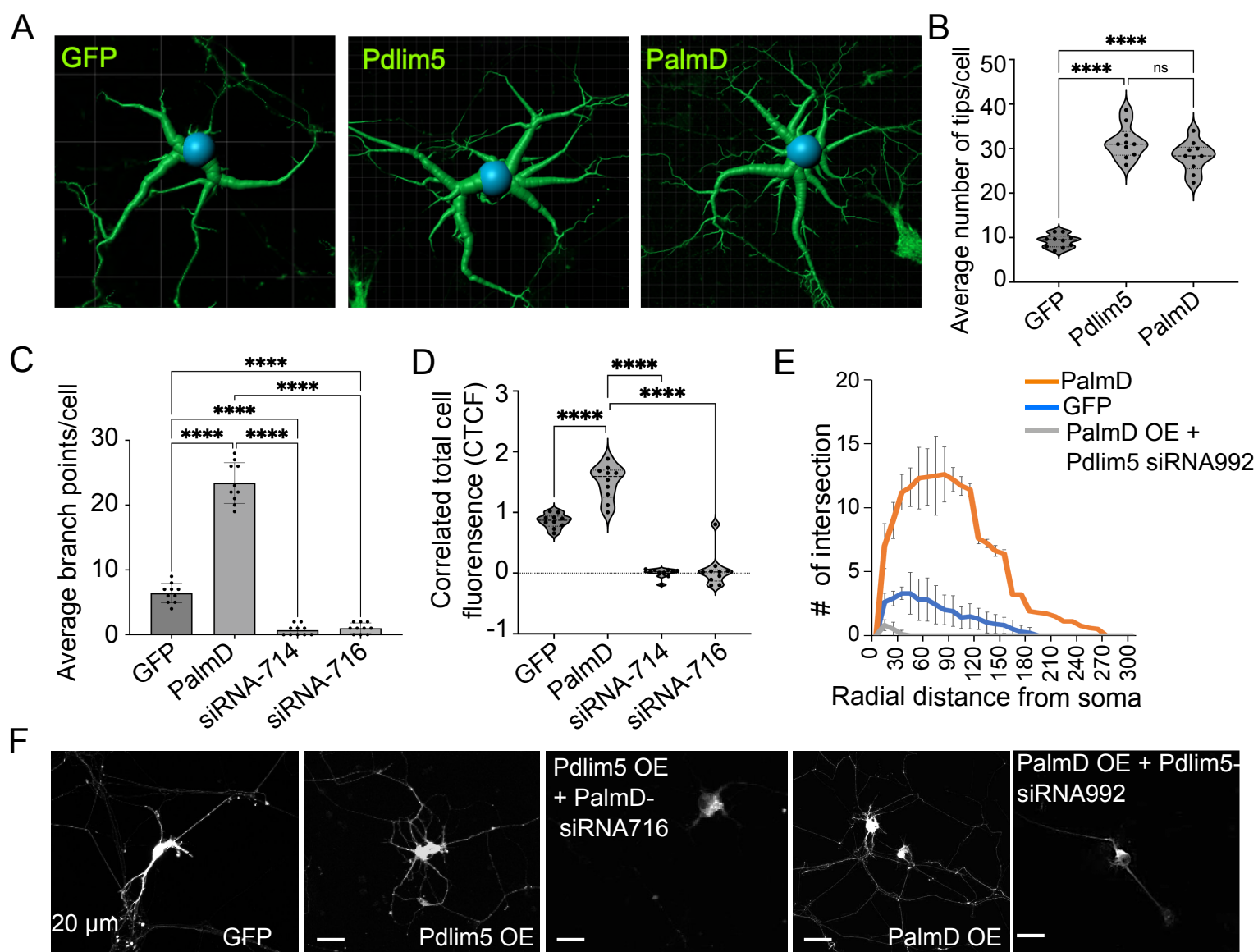

Supplement: Supplementary file 5 [file Image_4.pdf]
