# Supplementary material for: Role of a Pdlim5:PalmD complex in directing dendrite morphology
Source: Front Cell Neurosci. 2024 Feb 13;18:1315941. doi: 10.3389/fncel.2024.1315941 (PMC10896979; doi:10.3389/fncel.2024.1315941)
Supplement: Supplementary file 3 [file Image_2.pdf]

Figure S2: Selected Pdlim5 associated candidates with known cytoskeletal functions

Pdlim5 Y2H screen: Selected Prey (Gene Product) Findings and Roles

PalmD	Linker of cytoskeleton to the plasma membrane.
Actn4	alpha-Actinin4, cross-linker of actin microfilaments
Macf1	Facilitates actin-MT interaction
Nin	Anchoring minus-end MTs.
Palld	Component of microfilaments. Cell shape control.
Pcnt	MT nucleation. Interacts with gamma-tubulin.
Sorbs2	Potential link between Abl family kinases and actin.
Specc1l	Actin organization and MT stabilization.
Sptbn2	Beta-III Spectrin. Actin binding cytoskeletal scaffold.
Sptbn4	Beta-IV Spectrin. Actin binding cytoskeletal scaffold.
Vps18	Vesicle trafficking endosome/ lysosome.
